# Early warning of hand, foot, and mouth disease transmission: A modeling study in mainland, China

**DOI:** 10.1371/journal.pntd.0009233

**Published:** 2021-03-24

**Authors:** Jia Rui, Kaiwei Luo, Qiuping Chen, Dexing Zhang, Qinglong Zhao, Yanhong Zhang, Xiongjie Zhai, Zeyu Zhao, Siyu Zhang, Yuxue Liao, Shixiong Hu, Lidong Gao, Zhao Lei, Mingzhai Wang, Yao Wang, Xingchun Liu, Shanshan Yu, Fang Xie, Jia Li, Ruoyun Liu, Yi-Chen Chiang, Benhua Zhao, Yanhua Su, Xu-Sheng Zhang, Tianmu Chen

**Affiliations:** 1 State Key Laboratory of Molecular Vaccinology and Molecular Diagnostics, School of Public Health, Xiamen University, Xiamen City, Fujian Province, People’s Republic of China; 2 Hunan Provincial Center for Disease Control and Prevention, Changsha City, Hunan Province, People’s Republic of China; 3 Université de Montpellier, Montpellier, France; CIRAD, Intertryp, Montpellier, France; IES, Université de Montpellier-CNRS, Montpellier, France; 4 Medical Insurance Office, Xiang’an Hospital of Xiamen University, Xiamen City, Fujian Province, People’s Republic of China; 5 Jockey Club School of Public Health and Primary Care, The Chinese University of Hong Kong, Hong Kong Special Administrative Region, People’s Republic of China; 6 Jilin Provincial Center for Disease Control and Prevention, Changchun City, Jilin Province, People’s Republic of China; 7 Yunxiao County Center for Disease Control, Zhangzhou City, Fujian Province, People’s Republic of China; 8 Longde County Center for Disease Control, Guyuan City, the Ningxia Hui Autonomous Region, People’s Republic of China; 9 Shenzhen Centers for Disease Control and Prevention, Shenzhen City, Guangdong Province, People’s Republic of China; 10 Xiamen City Center for Disease Control and Prevention, Shenzhen City, Fujian Province, People’s Republic of China; 11 Public Health England, London, United Kingdom; Chengde Medical University, CHINA

## Abstract

**Background:**

Hand, foot, and mouth disease (HFMD) is a global infectious disease; particularly, it has a high disease burden in China. This study was aimed to explore the temporal and spatial distribution of the disease by analyzing its epidemiological characteristics, and to calculate the early warning signals of HFMD by using a logistic differential equation (LDE) model.

**Methods:**

This study included datasets of HFMD cases reported in seven regions in Mainland China. The early warning time (week) was calculated using the LDE model with the key parameters estimated by fitting with the data. Two key time points, “epidemic acceleration week (EAW)” and “recommended warning week (RWW)”, were calculated to show the early warning time.

**Results:**

The mean annual incidence of HFMD cases per 100,000 per year was 218, 360, 223, 124, and 359 in Hunan Province, Shenzhen City, Xiamen City, Chuxiong Prefecture, Yunxiao County across the southern regions, respectively and 60 and 34 in Jilin Province and Longde County across the northern regions, respectively. The LDE model fitted well with the reported data (*R*^2^ > 0.65, *P* < 0.001). Distinct temporal patterns were found across geographical regions: two early warning signals emerged in spring and autumn every year across southern regions while one early warning signals in summer every year across northern regions.

**Conclusions:**

The disease burden of HFMD in China is still high, with more cases occurring in the southern regions. The early warning of HFMD across the seven regions is heterogeneous. In the northern regions, it has a high incidence during summer and peaks in June every year; in the southern regions, it has two waves every year with the first wave during spring spreading faster than the second wave during autumn. Our findings can help predict and prepare for active periods of HFMD.

## Author summary

Hand, foot, and mouth disease (HFMD), a global infectious disease, has a high disease burden in China. This study aimed to explore the temporal and spatial distribution of the disease by analyzing its epidemiological characteristics and to calculate the early warning signals of HFMD by using a logistic differential equation (LDE) model. Our findings showed that 1) the HFMD disease burden in China remained high and HFMD incidence in the southern region was higher than that in the northern region; and 2) the HFMD early warnings times across China were heterogeneous. One high incidence period occurred in the northern regions during summer, peaking in June every year, while in the southern regions two waves emerged every year. Over the southern regions, the disease appeared to spread faster in the spring wave than in the autumn wave. These results provide important information for public health authorities such as the Centers for Disease Control and Prevention staff to predict and prepare for the onset of the high active periods of HFMD, especially by indicating the heterogeneous warning times of HFMD outbreak across different geographical regions in Mainland China.

## Introduction

Hand, food, and mouth disease (HFMD) has become a global infectious disease, and in recent years, the coxsackievirus (CV)-A16 subtype has been the major pathogen in China[1–3]. The most susceptible population is children under the ages of 5[4,5]. Intestinal fever, palm and foot rash, and oral herpes or ulcers are the common features of presentation, and the mortality rate can be as high as 1.8%[6–8]. Most patients have self-limiting symptoms, whereas some may experience complications, such as myocarditis, neurogenic pulmonary edema, and aseptic meningoencephalitis.

On April 19, 1957, J. H. Seddon of New Zealand submitted a report describing eight new cases of the childhood clinical disease, the first proposed cases of HFMD[9]; following which the epidemic of HFMD appeared in most parts of the world[10–12]. Although HFMD caused a severe epidemic in East Asia in the late 1990s[11,13], the first report of HFMD in China was in 1974[14–16], and outbreaks of HFMD have also been reported. After 2008, HFMD showed a tendency to spread, appearing even in various regions of China. HFMD was officially included in the national statutory Class C infectious disease surveillance in May 2008[17]. The current epidemic situation remains severe, with a persistently high and increasing incidence rate. Although there is already a vaccine against enterovirus 71 (EV71), another pathogen of HFMD [18,19], the vaccination rate is still relatively low as it is a second-class vaccine, and its use is optional [20]. Additionally, the vaccine’s capability of HFMD control and prevention still need further investigation. Early identification of severe diseases remains the key to successful treatment. It is therefore necessary to conduct in-depth research on the epidemiological characteristics of HFMD, which has important significance for the early warning of the disease.

Existing research methods for early warning of infectious diseases include time series models, GM (1,1) gray model; logistic differential equation (LDE) model[21], and ordinary differential equation models[22–31]. Our pilot study showed that the LDE model can be used to describe the epidemic characteristics of the outbreak periods of infectious diseases. It can also indicate the point at which the early epidemic speed changes from slow to fast. Thus, it is suitable for the simulation of epidemic characteristics in various epidemics of HFMD.

**Fig 1:**
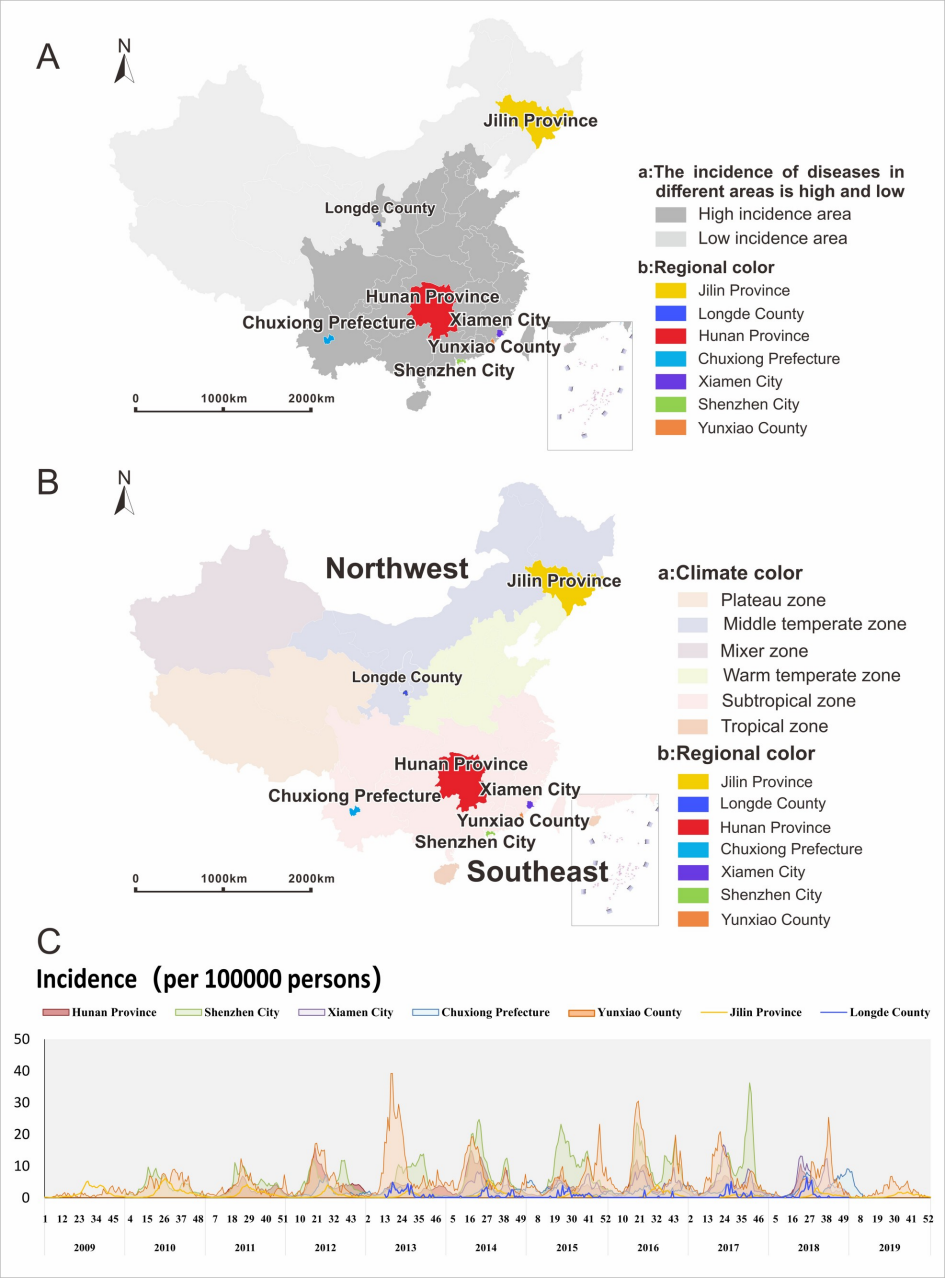
Geographical distribution and incidence distribution in Hunan Province, Jilin Province, Shenzhen City, Xiamen City, Chuxiong Prefecture, Yunxiao County, and Longde County. Fig 1A shows the locations and reported cases of in Hunan Province, Jilin Province, Shenzhen City, Xiamen City, Chuxiong Prefecture Yunxiao County, and Longde County in the map of mainland China. The dark grey regions include provinces with severe HFMD outbreaks every year. China can be roughly divided into two parts according to its humidity and temperature: the drier and colder part in the northwest and the more humid and warmer part in the southeast (Fig 1B). Five of the seven regions belong to the subtropical zone and two belongs to the mid-temperate zone. The southeast part includes tropical, subtropical and warm temperate zones; the northwest part includes middle temperate zone, the plateau zone and the middle and warm mixture zone. Fig 1C shows the time series of incidence in Hunan Province, Jilin Province, Shenzhen City, Xiamen City, Chuxiong Prefecture, Yunxiao County, and Longde County. The map depicted in this figure was taken from Wikimedia Commons https://commons.wikimedia.org/wiki/Atlas_of_the_People%27s_Republic_of_China#/media/File:China_Hunan.svg.

This study collected the HFMD case data from 2009 to 2019 of seven regions (Hunan Province, Jilin Province, Shenzhen City, Xiamen City, Chuxiong Prefecture, Longde County, and Yunxiao County) and carried out a space-time early warning research analysis based on the LDE model. We chose these seven regions because: 1) they were designated from high and low incidence areas based on a national study conducted by Zhao et al [32]; 2) they have a high population densities and their distribution covers the different regions of the northern and southern China (north, east and south), which are high-risk HFMD areas; and 3) Geographically, China spans multiple climatic zones, and the incidence of HFMD in each province has a regional heterogeneity due to the large socio-economic and climatic differences. Therefore, our study selected the seven areas to carry out predictive and early warning analysis of HFMD outbreaks (Fig 1).

## Methods

### Data collection

We collected data on HFMD from each province in China from 2013 to 2017 using the National Population Health Science Data Center (http://www.phsciencedata.cn/Share/index.jsp). Our research aimed to further study the detailed HFMD outbreak features using selected HFMD data, in weeks, from two provinces (Hunan Province and Jilin Province), two cities (Shenzhen City and Xiamen City), one autonomous prefecture (Chuxiong Prefecture), and two counties (Longde County and Yunxiao County) from the six temperature zones in China. Among them, Hunan Province, Shenzhen City, Xiamen City, Chuxiong Prefecture and Yunxiao County are located in southern China, while Jilin Province and Longde County are located in northern China. Furthermore, Shenzhen City and Xiamen City with their six districts, Chuxiong Prefecture, and Yunxiao County are economically developed areas with relatively dense populations in several major provinces on the southeast coast, and all are in the subtropical monsoon climate region.

In this study, we used the well-established reported dataset for HFMD cases from Hunan Province (and from its 14 cities or prefectures) from the 1st week of 2011 to the 52nd week of 2018; in Jilin Province (and its nine cities), we used data reported from the 1st week of 2009 to the 52nd week of 2019; in Shenzhen City (and its six districts), we used data reported from the 1st week of 2010 to the 52nd week of 2017; in Xiamen City (and its six districts), we used data reported from the 7th week of 2014 to the 52nd week of 2018; in Chuxiong Prefecture, we used data reported from the 1st week of 2012 to the 7th week of 2019; in Longde County, we used data reported from the 1st week of 2013 to the 52nd week of 2018; and in Yunxiao County, we used data reported from the 1st week of 2009 to the 52nd week of 2019. The extracted date for each case included date of illness onset, while the case types included clinically diagnosed and laboratory confirmed cases. All the cases were obtained from the Chinese Disease Prevention and Control Information System.

### LDE model

The LDE model was first proposed by Verhust in 1845 to describe the population’s self-growth characteristics of the ordinary differential equation (ODE) model[33]. The model differential equation is as follows:

$$\frac{dn}{dt}=kn(1-\frac{n}{N})$$ (1)

where *dn/dt* is the rate of change of cumulative case *n* of infectious diseases at time *t*, *k* is the model correlation coefficient, and *N* is the cumulative case limit of infectious diseases. The *dn/dt = kn* in the equation is a Malthusian model, whose epidemiological significance is that cumulative cases increase exponentially over time. 1*-n/N* is an adjustment to the Malthusian model; the value of 1*-n/N* ranges from 0 to 1. When *n* is small, 1*-n/N* tends toward 1. The logistic model at this time is close to the Malthusian model. When *n* is gradually increased, they move closer to *N*, and 1*-n/N* tends towards zero. Therefore, 1*-n/N* has epidemiological significance. With the epidemic, due to the establishment of the population immune barrier (the near saturation level of the pathogens in the host population), the epidemic will tend to cease, and new cases will gradually decrease, then the epidemic ends. Therefore, the logistic model curve is “*S*” shaped, and the disease development is from “slow-fast-slow”. The general solution of Eq 1 is:

$$n=\frac{N}{1+e^{-kt-c}}$$ (2)

where *c* is a constant.

In the LDE model, the curve change speed is changed from the slower and faster turning point. To calculate the turning point, we set the third derivative of Eq 2 equal to 0.

$$\frac{d^{3}n}{dt^{3}}=\frac{Nk^{3}e^{-(kt+c)}(1-4ke^{-(kt+c)}+e^{-2(kt+c)})}{{\left(1+e^{-(kt+c)}\right)}^{4}}=0$$ (3)

**Fig 2:**
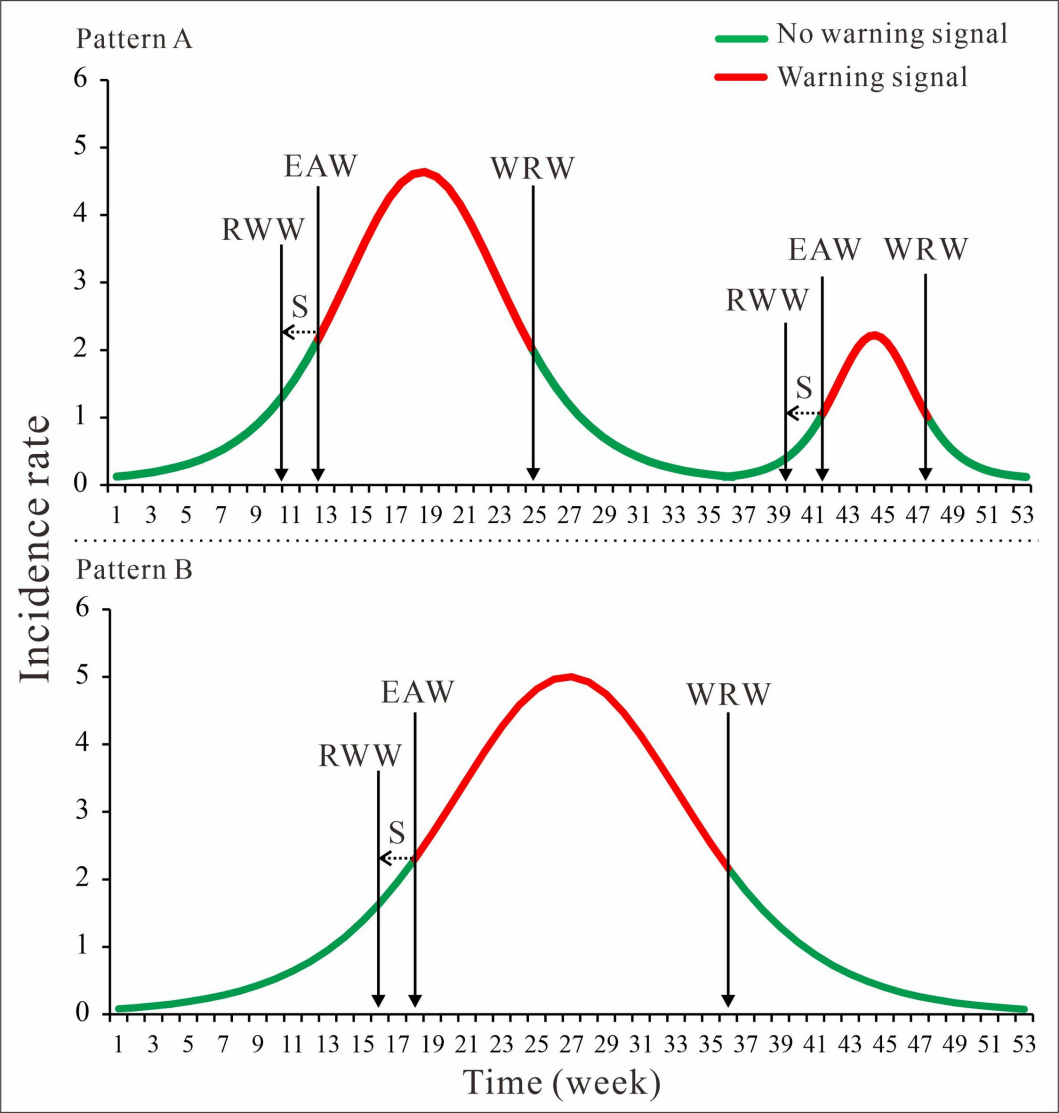
Diagram of method to determine early warning week of HFMD in epidemic cycle. (*S* is the standard deviation; EAW is epidemic acceleration week; RWW is recommended warning week; WRW is warning removed week).

The answer is:

$$t=\frac{-c\pm 1.317}{k}$$ (4)

where *t*_1_ = $\frac{-c-1.317}{k}$ is the abscissa corresponding to the inflection point where the curve change speed is slower to faster in the LDE model, that is the “epidemic acceleration week (EAW)”. At the same time, calculate the average value and standard deviation (*S*) of the "EAW" of each HFMD epidemic cycle in each region were calculated. According to previous research[21], *S* is usually over 2 weeks. Considering that intervention and implementation of the HFMD epidemic in the seven selected region requires considerable time each year, waiting for the epidemic to develop EAW to give a signal of early warning will lead to poor control effects. Therefore, this study used the time called “recommended warning week (RWW)” when the EAW advances, the *S* is the time of early warning, as shown in Fig 2, RWW = EAW-2, the units of RWW and EAW are both in weeks.

And *t*_2_ = $\frac{-c+1.317}{k}$ is the abscissa corresponding to the inflection point where the curve change speed is faster to slower in the LDE model, that is the “warning removed week (WRW)”.

### Mathematical simulation and data processing methods

This study first analyzed the periodicity of the number of cases of HFMD from the 1st week of 2011 to the 20th week of 2018 in Hunan Province. The nearly 8–year data were divided into several epidemic cycles according to the characteristics of their distribution peaks. Then, the number of HFMD cases in Jilin Province, Shenzhen City, Xiamen City, Chuxiong Prefecture, Longde County, and Yunxiao County was divided according to the periodicity.

The software program used in the simulation of this model was Berkeley Madonna 8.3.18. Microsoft Excel (2020) was used for the entry and management of related data and related mapping. The differential equation solving method was used with a fourth-order Runge-Kutta method with a tolerance of 0.001. The criterion for the goodness of curve fitting was the least root mean square of the simulated data and the reported data. Spatial distribution analysis was conducted to determine the incidence of HFMD in the seven regions.

## Results

### Distribution of HFMD in china

**Fig 3:**
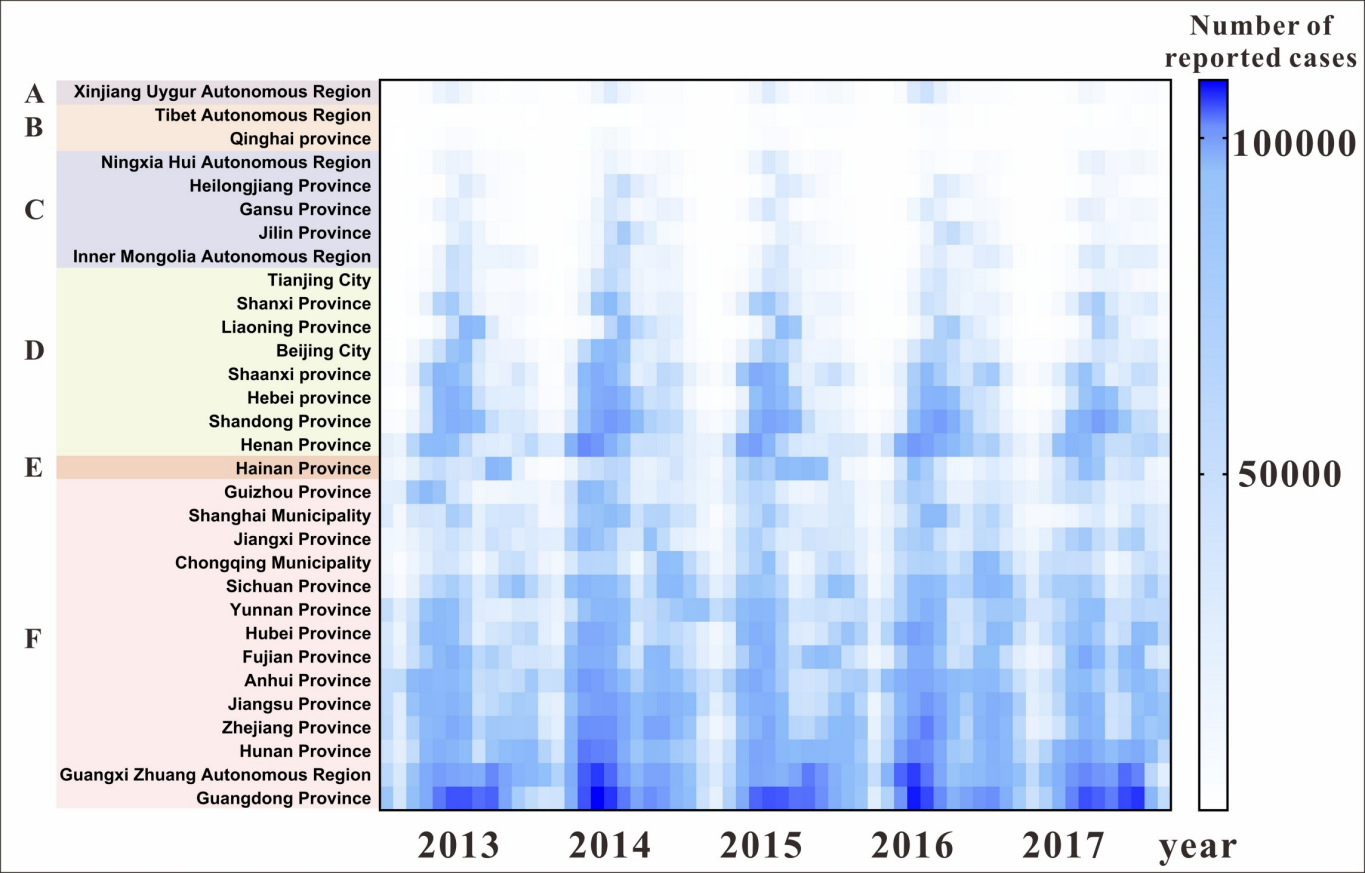
Distribution of Hand, Foot and Mouth Disease in Six Temperature Zones in China. (A: Mixer Zone; B: Plateau Zone; C: Middle Temperature Zone; D: Warm Temperature Zone; E: Subtropical Zone; F: Tropical Zone).

The heat map of Fig 3 showed a higher number of yearly incidences in the southern region provinces (in different temperature zones) than those in the northern region. While the southern region exhibited obvious bimodal distribution characteristics, the northern region had a mainly unimodal distribution. The peak incidence in the northern region was mostly in June each year, but in the southern region, it alternated in time between spring vs. summer and autumn vs. winter.

### Epidemic characteristics of HFMD in the seven selected regions

In high HFMD incidence areas, the annual incidence of HFMD cases per 100,000 population was 218, 360, 223, 124, 359 in Hunan Province (*N* = 1,195,411 cases between 2011 and 2018), Shenzhen City (*N* = 360,622 cases between 2010 and 2017), Xiamen City (*N* = 43,666 cases between 2014 and 2018), Chuxiong Prefecture (*N* = 27,108 cases between 2012 and 2019), and Yunxiao County (*N* = 15,371 cases between 2009 and 2019), respectively. In relatively low HFMD incidence areas, the incidences were 60 and 34 in Jilin Province (N = 160,296 cases between 2009 and 2019) and Longde County (*N* = 326 cases between 2013 and 2018), respectively (Fig 1C).

**Fig 4:**
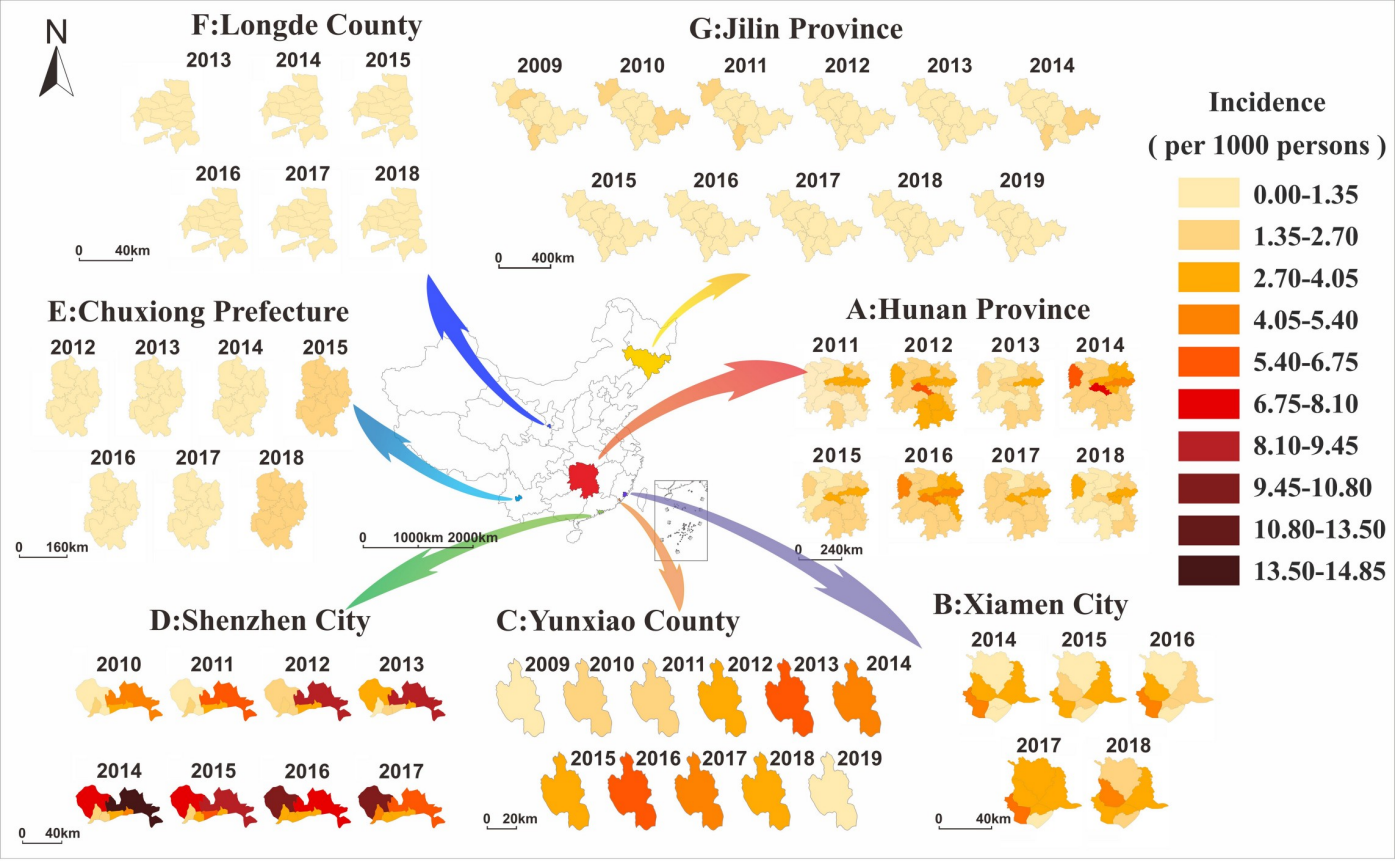
The temporal and spatial distribution of HFMD in Hunan Province, Jilin Province, Shenzhen City, Xiamen City, Chuxiong Prefecture, Yunxiao County, and Longde County. The map depicted in this figure was taken from Wikimedia Commons (http://commons.wikimedia.org/wiki/Main_Page). (A: Hunan Province; B: Xiamen City; C: Yunxiao County; D: Shenzhen City; E: Chuxiong Prefecture; F: Longde County; G: Jilin Province).

**Fig 5:**
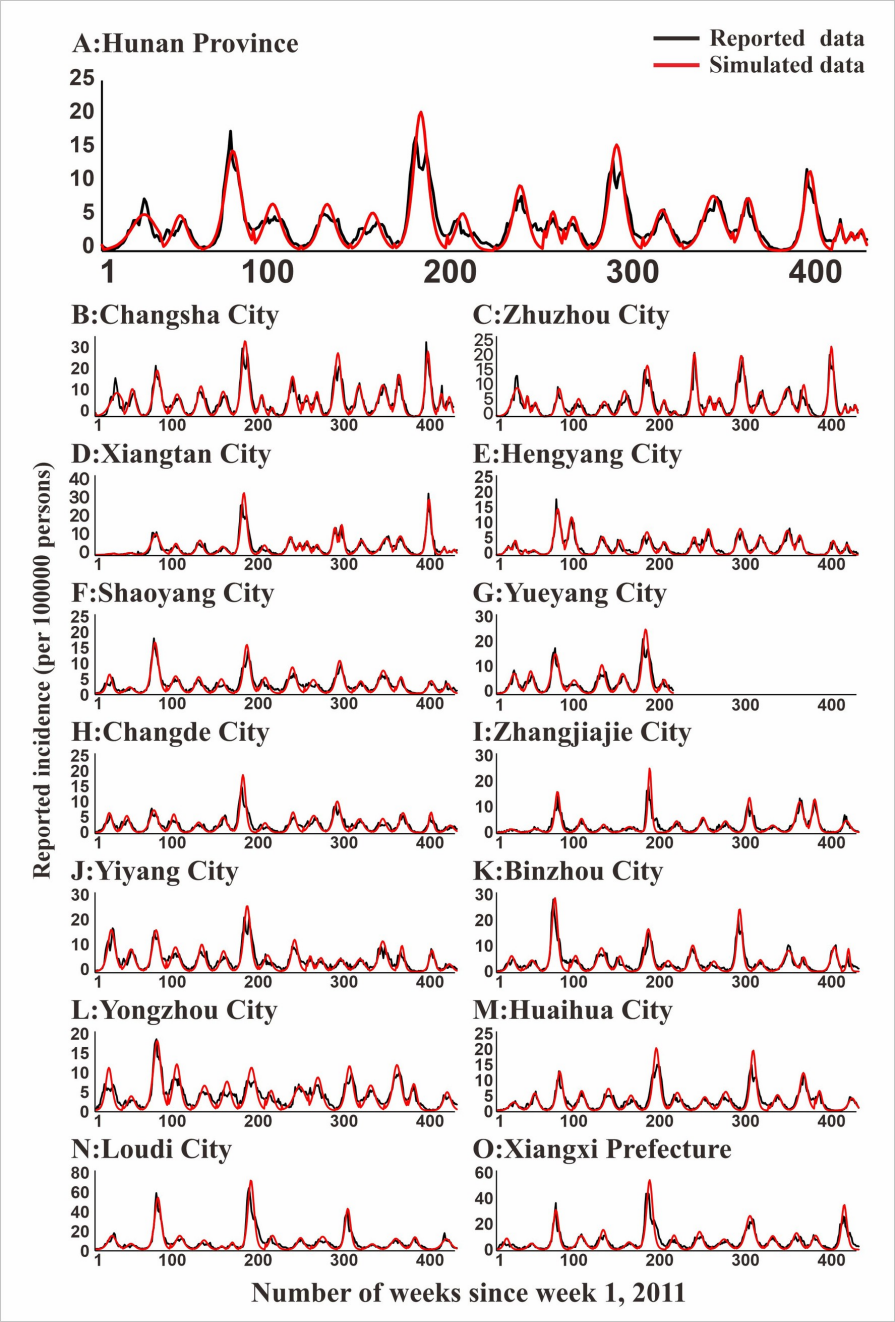
Epidemics of HFMD and LDE model fittings within Hunan Province and its 14 regions. (A: Hunan Province; B: Changsha City; C: Zhuzhou City; D: Xiangtan City; E: Hengyang City; F: Shaoyang City; G: Yueyang City; H: Changde City; I: Zhangjiajie City; J: Yiyang City; K: Chenzhou City; L: Yongzhou City; M: Huaihua City; N: Loudi City; O: Xiangxi Prefecture).

The HFMD incidence trend in the seven regions and their sub-regions are shown in Fig 4, which also depicts the HFMD incidences from 2011 to 2018 in 14 cities and prefectures in Hunan Province, and these incidences changed yearly. In 2014, the incidence of HFMD was generally the highest in Hunan Province, followed by a significant reducing trend. Among these, the incidence rates of Loudi City, Changsha City, and Xiangxi Prefecture were high, while the incidence rates of Zhangjiajie City, Changde City, and Hengyang City were at a low level (Figs 4 and 5). While the incidence in Shenzhen City was the highest, the incidence in Longde County was the lowest. The increasing depth of color, representing the number of the incidence per 1000 persons in Shenzhen City and Xiamen City, indicates that the incidence of HFMD in these areas has become more and more serious in recent years. The color change is slightly fluctuant in Chuxiong Prefecture, although the incidence of HFMD in the area has a slightly ascending trend from 2012 to 2018 in the area.

### Model fitting

**Fig 6:**
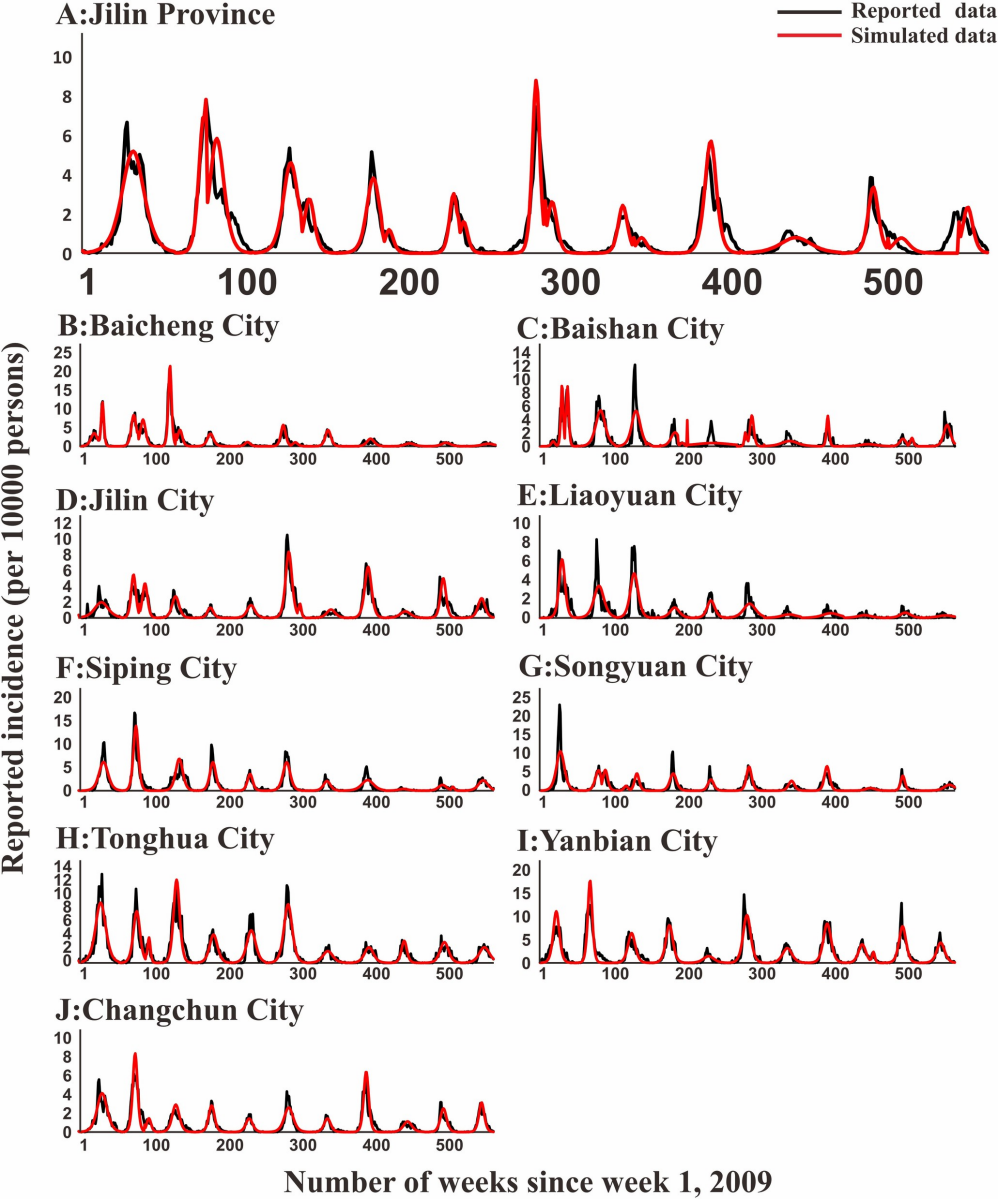
Epidemics of HFMD and LDE model fittings within Jilin Province and its 9 regions. (A: Jilin Province; B: Baicheng City; C: Baishan City; D: Jilin City; E: Liaoyuan City; F: Siping City; G: Songyuan City; H: Tonghua City; I: Yanbian City; J: Changchun City).

**Fig 7:**
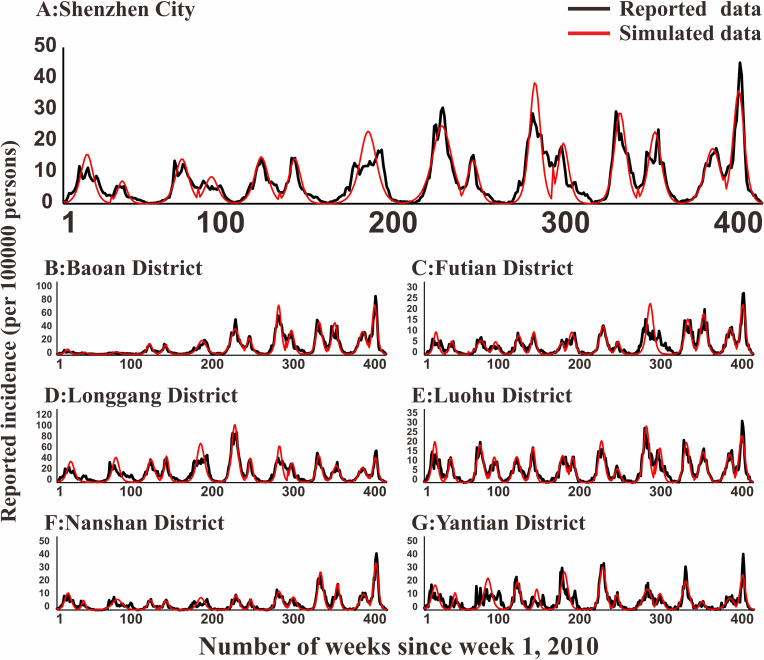
Epidemics of HFMD and LDE model fittings within ShenZhen City and its 6 districts. (A: Shenzhen City; B: Baoan District; C: Futian District; D: Longgang District; E: Luohu District; F: Nanshan District; G: Yantian District).

**Fig 8:**
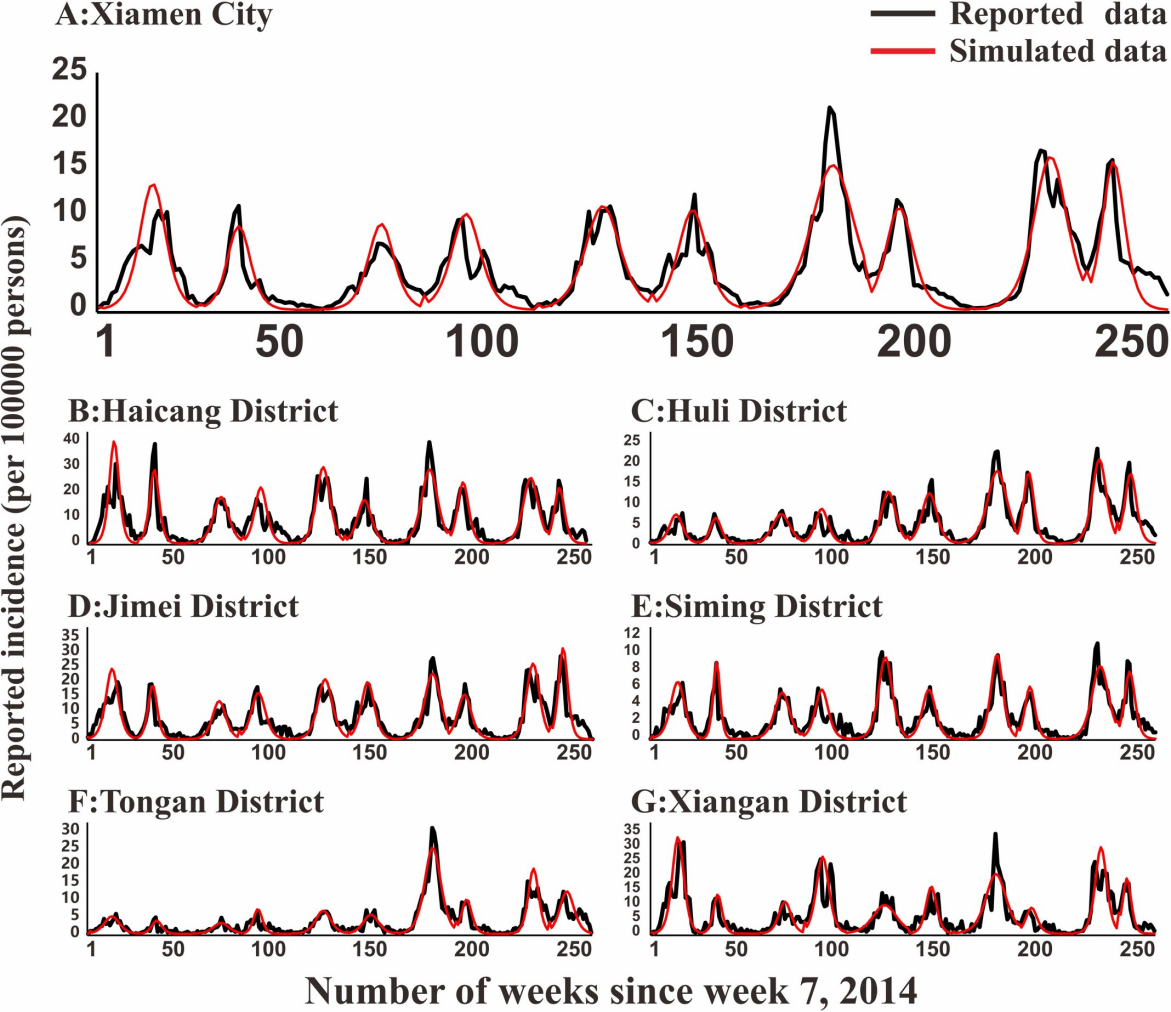
Epidemics of HFMD and LDE model fittings within Xiamen City and its 6 districts. (A: Xiamen City; B: Haicang District; C: Huli District; D: Jimei District; E: Simin District; F: Tongan District; G: Xiangan District).

**Fig 9:**
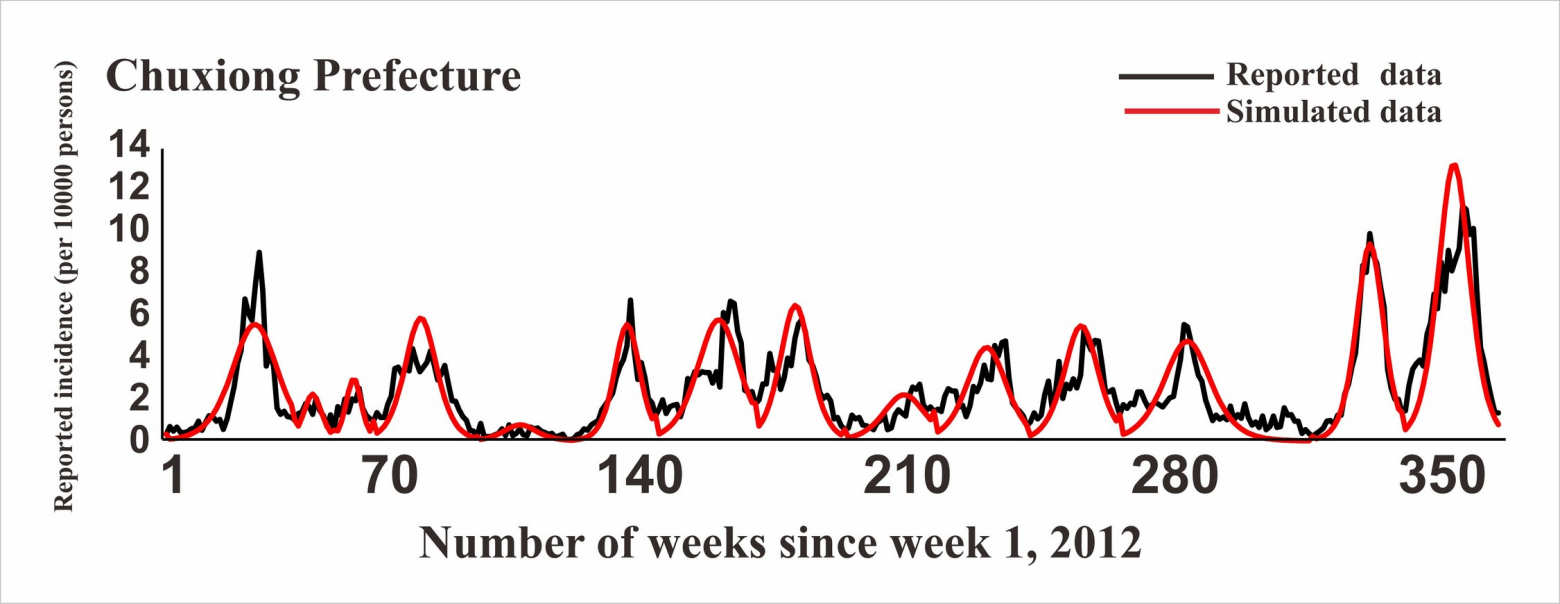
Epidemics of HFMD and LDE model fittings within Chuxiong Prefecture.

**Fig 10:**
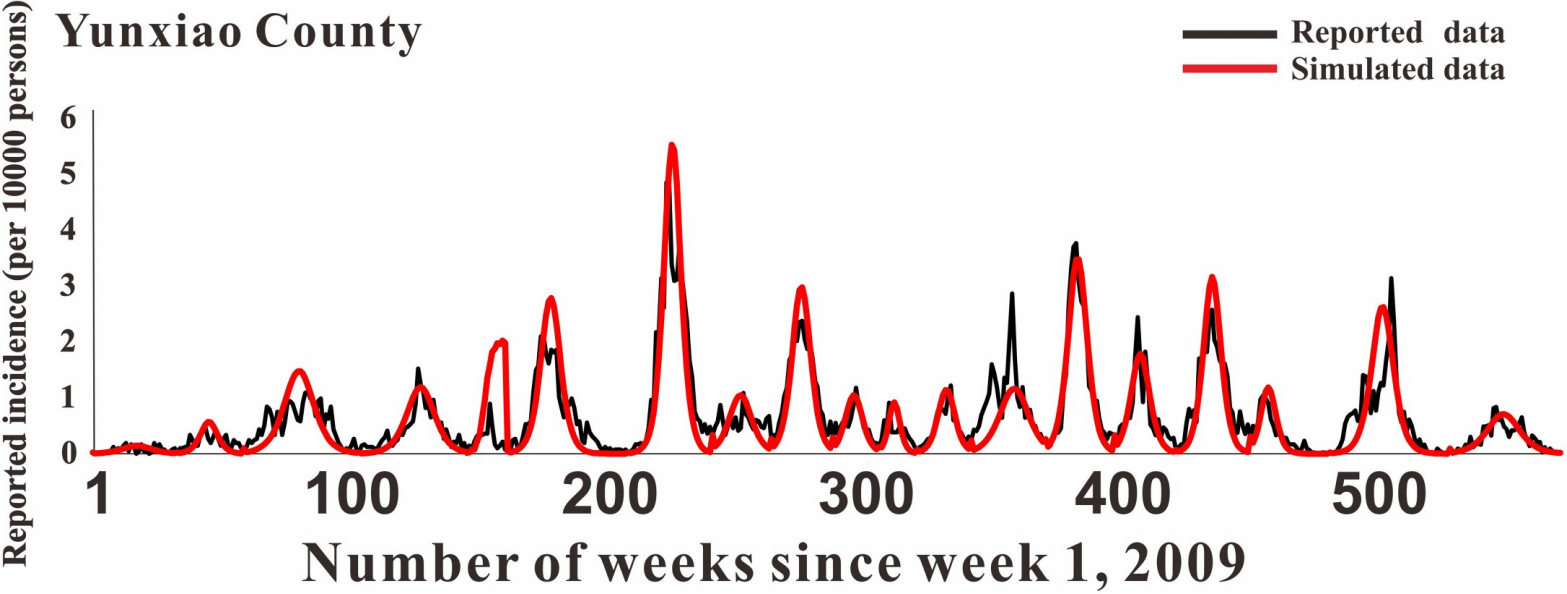
Epidemics of HFMD and LDE model fittings within Yunxiao County.

**Fig 11:**
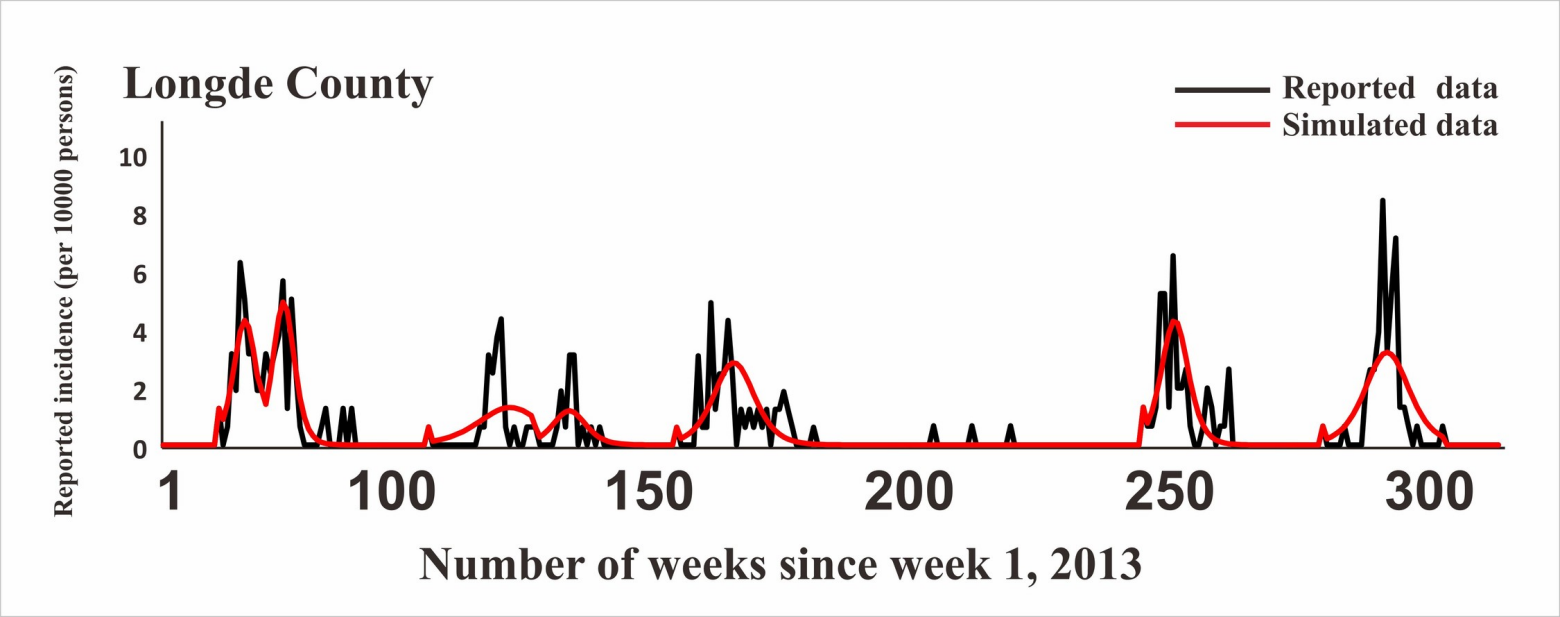
Epidemics of HFMD and LDE model fittings within Longde County.

In Fig 5, the fitting effect of the LDE model of Hunan Province and its 14 cities or prefectures and the reported data were shown, while Figs 6–11 showed the fitting effect of the LDE model and reported data of Jilin Province and its nine cities, Shenzhen City and its six districts, Xiamen City and its six districts, Chuxiong Prefecture, Yunxiao County, and Longde County. Table 1 showed that the result of simulated data was very close to the reported data, with a well-fitted effect (*R*^*2*^ in the seven regions were close to 1, and *P* < 0.05). The parameters (*k* and c) for the model fitting of the seven regions in the spring and autumn are shown in Table 2.

**Table 1:** Fitting results of LDE model with the selected data.

Region	*R*^2^	*P*
Hunan Province	0.994	<0.001
Changsha City	0.996	<0.001
Zhuzhou City	0.995	<0.001
Xiangtan City	0.996	<0.001
Hengyang City	0.996	<0.001
Shaoyang City	0.993	<0.001
Yueyang City	0.993	<0.001
Changde City	0.990	<0.001
Zhangjiajie City	0.991	<0.001
Yiyang City	0.993	<0.001
Chenzhou City	0.994	<0.001
Yongzhou City	0.990	<0.001
Huaihua City	0.991	<0.001
Loudi City	0.994	<0.001
Xiangxi State	0.991	<0.001
Jilin Province	0.820	<0.001
Baicheng City	0.893	<0.001
Baishan City	0.704	<0.001
Jilin City	0.800	<0.001
Liaoyuan City	0.700	<0.001
Siping City	0.831	<0.001
Songyuan City	0.704	<0.001
Tonghua City	0.870	<0.001
Yanbian City	0.837	<0.001
Changchun City	0.860	<0.001
Shenzhen City	0.821	<0.001
Baoan District	0.885	<0.001
Futian District	0.710	<0.001
Longgang District	0.794	<0.001
Luohu District	0.835	<0.001
Nanshan District	0.858	<0.001
Yantian District	0.652	<0.001
Xiamen City	0.821	<0.001
Haicang District	0.768	<0.001
Huli District	0.842	<0.001
Jimei District	0.783	<0.001
Simin District	0.812	<0.001
Tongan District	0.849	<0.001
Xiangan District	0.705	<0.001
Chuxiong Prefecture	0.787	<0.001
Yunxiao County	0.758	<0.001
Longde County	0.578	<0.001

**Table 2:** Parameters calculated by logistic model to fit the HFMD data in seven areas in China.

Region	k		c	
	Median	range	Median	range
Hunan Province	0.377	0.3381–0.5073	84.822	44.1554–115.229
Changsha City	0.384	0.3494–0.5123	149.072	79.0229–218.122
Zhuzhou City	0.500	0.3526–0.631	55.390	32.3065–139.8935
Xiangtan City	0.446	0.3447–0.6214	54.973	29.4977–112.4975
Hengyang City	0.425	0.3724–0.4728	58.517	30.4757–91.9574
Shaoyang City	0.373	0.3547–0.4085	58.857	46.7393–104.806
Yueyang City	0.367	0.3386–0.4085	91.536	71.6499–166.7123
Changde City	0.392	0.3349–0.4483	58.561	46.0958–71.2656
Zhangjiajie City	0.411	0.3471–0.4911	48.103	31.6473–108.198
Yiyang City	0.378	0.3355–0.4231	86.779	68.548–143.065
Chenzhou City	0.394	0.358–0.4522	58.668	44.6917–116.5575
Yongzhou City	0.345	0.313–0.3837	101.847	58.1182–126.765
Huaihua City	0.384	0.3604–0.4319	59.687	50.1038–112.325
Loudi City	0.376	0.3209–0.4421	137.094	76.785–172.0178
Xiangxi Prefecture	0.393	0.3608–0.4582	122.616	93.4807–234.997
Jilin Province	0.361	0.155–0.5429	42.465	19.754–125.067
Baicheng City	0.428	0.1781–0.8298	40.646	9.7287–125.067
Baishan City	0.528	0.0575–1.0515	23.641	2.7253–493.9382
Jilin City	0.322	0.1591–0.8342	35.260	8.8442–100.945
Liaoyuan City	0.207	0.1287–0.3726	26.134	7.2476–128.792
Siping City	0.302	0.1849–0.7578	43.578	4.466–128.792
Songyuan City	0.376	0.2094–0.558	38.711	9.4231–155.805
Tonghua City	0.283	0.2084–0.7816	54.861	19.6928–155.805
Yanbian City	0.305	0.2093–0.7858	90.803	12.1509–142.569
Changchun City	0.346	0.2165–0.5036	31.015	11.7535–76.8886
Shenzhen City	0.431	0.3743–0.4621	165.879	133.7050–297.3690
Baoan District	0.442	0.3480–0.4782	298.938	103.7016–498.3513
Futian District	0.389	0.3655–0.5322	86.434	56.0791–167.3740
Longgang District	0.394	0.3421–0.4868	431.689	316.7575–520.8960
Luohu District	0.419	0.3735–0.4707	171.547	123.5545–204.0740
Nanshan District	0.413	0.3627–0.5198	126.033	68.3902–148.9630
Yantian District	0.387	0.3507–0.4930	204.217	103.2509–243.7353
Xiamen City	0.455	0.3778–0.5081	99.029	85.9978–129.9125
Haicang District	0.461	0.3910–0.5927	173.200	160.6130–243.1945
Huli District	0.464	0.3902–0.5277	105.170	62.9884–133.5048
Jimei District	0.458	0.4240–0.5278	166.254	123.9878–190.5508
Siming District	0.462	0.4243–0.5842	49.533	0.5842–73.1573
Tongan District	0.408	0.3869–0.5956	57.187	37.0798–111.2725
Xiangan District	0.523	0.4271–0.6057	121.132	78.9997–212.3233
Chuxiong Prefecture	0.330	0.2551–0.3998	71.048	30.4225–93.6741
Yunxiao County	0.413	0.2386–0.7143	1.723	0.2160–4.9578
Longde County	0.394	0.1997–0.6041	31.550	10.509–42.2944

### Determination of the early warning week in the seven selected regions

**Fig 12:**
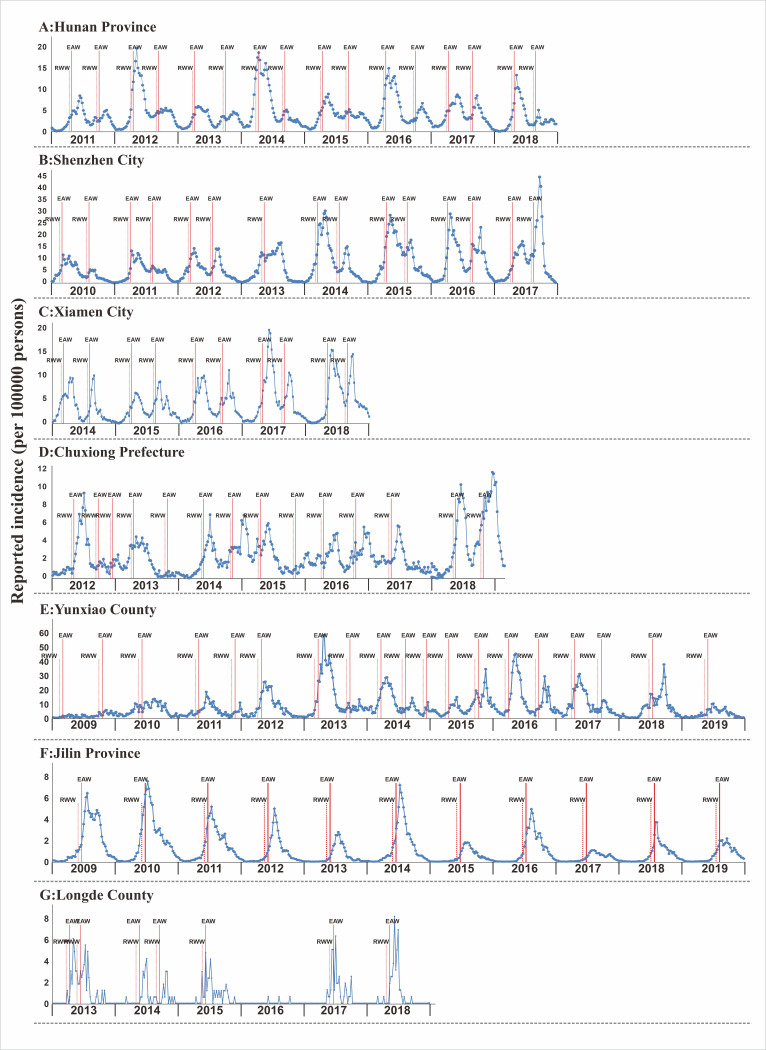
“EAW” and “RWW” of HFMD in spring and autumn in Hunan Province, Jilin Province, Shenzhen City, Xiamen City, Chuxiong Prefecture, Yunxiao County, and Longde County based on the weekly data. (A: Hunan Province; B: Shenzhen City; C: Xiamen City; D: Chuxiong Prefecture; E: Yunxiao County; F: Jilin Province; G: Longde County).

As shown in Fig 12, there were two HFMD incidence peaks (in the spring and autumn) in Hunan Province, Shenzhen City, Xiamen City, Chuxiong Prefecture, and Yunxiao County were observed, but there was only one incidence peak (in summer) in the two regions (Jilin Province and Longde County). For the data of the seven regions, the EAW calculated by the LDE model was distributed at the accelerating inflection point of the upward phase of each year. However, by the time the EAW appeared, the epidemic situation had risen to a higher level, meaning that the indicator warning appeared to be delayed; therefore, based on the EAW result, we further calculated the RWW using the calculation formula.

### Seasonal distribution characteristics of HFMD epidemic in the seven selected regions

**Fig 13:**
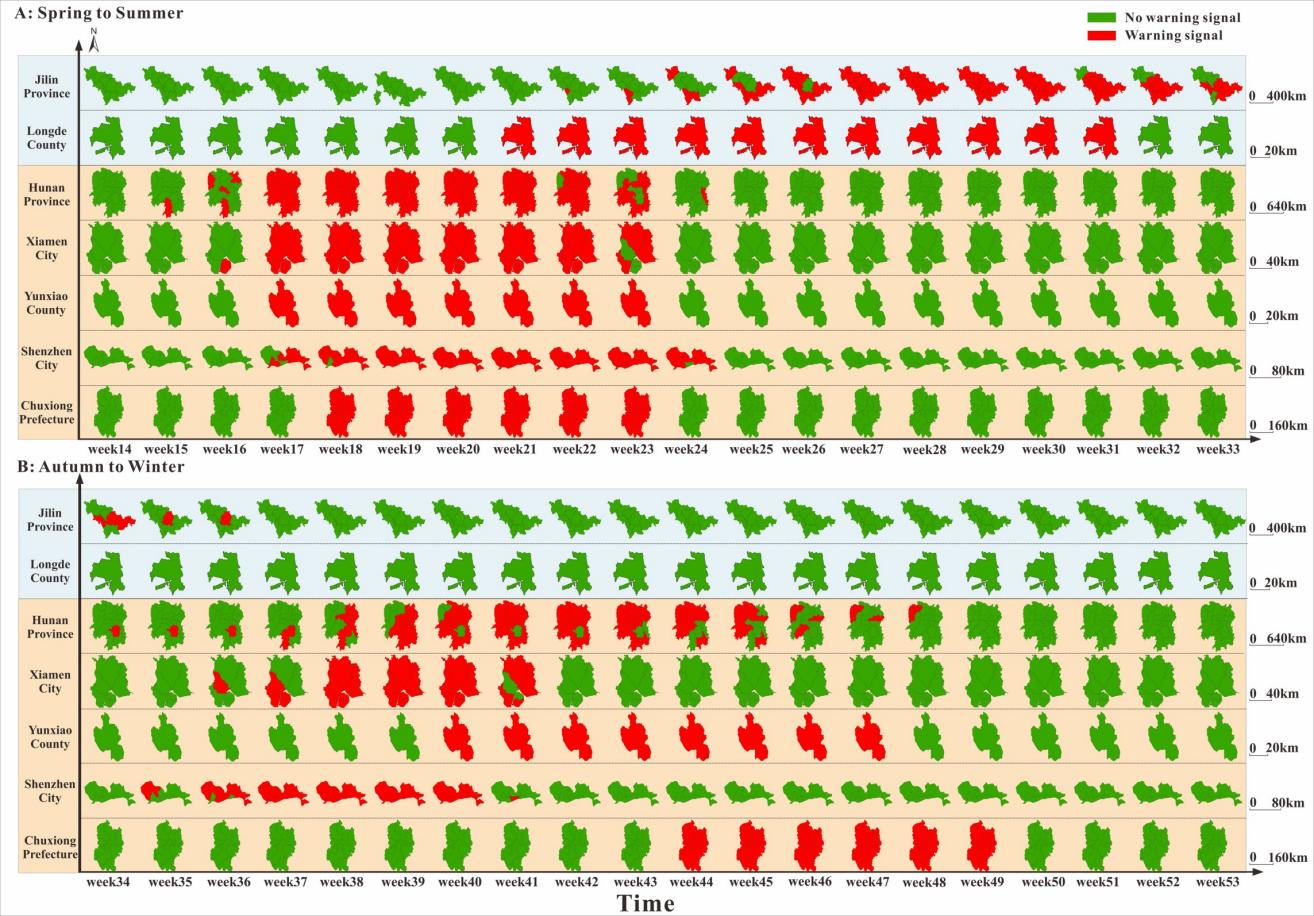
Time and space warning map in Hunan Province, Jilin Province, Shenzhen City, Xiamen City, Chuxiong Prefecture, Yunxiao County, and Longde County. The warning signals in this figure were median early warning week of each area. (A: Spring to Summer; B: Autumn to Winter; Red signals represent that the epidemic is in the accelerated period and indicate the beginning of early warning, and green signals represent that the epidemic is in the deceleration period, meaning that the early warning can be subsequently relieved.) The map depicted in this figure was taken from Wikimedia Commons https://commons.wikimedia.org/wiki/Atlas_of_the_People%27s_Republic_of_China#/media/File:China_Hunan.svg.

Fig 13 showed the median early warning week of Hunan Province, Jilin Province, Shenzhen City, Xiamen City, Chuxiong Prefecture, Yunxiao County, and Longde County by fitting the incidence data of each region with the data range being six years (2011–2018), eight years (2010–2017), five years (2014–2018), seven years (2012–2018), 11 years (2009–2019), 11 years (2009–2019), and six years (2013–2018) respectively. The range of weeks for showing the median of EWW of the seven regions is shown in S1 Table in the supplementary materials. From Fig 13A, the first HFMD epidemic occurred in the 15th week in Hunan Province, followed by Xiamen City in the 16th week, Shenzhen City and Yunxiao County in the 17th week, Chuxiong Prefecture in the 18th week, Longde County in the 21st week, and finally Jilin Province in the 22nd week. However, different from the time in the five regions in the mid-temperate zone, the time period spanned by all the nine cities in Jilin Province was longer; the duration of the epidemic in Jilin Province and Longde County was more than 10 weeks, while the duration of the epidemic in the five regions in the mid-temperate zone was 10weeks or less. Fig 13B (Autumn to Winter) shows that the epidemic occurred in the 34th week in Hunan Province and that after seven weeks, all 14 cities (prefectures) in Hunan Province had all had the HFMD outbreaks. However, by the 40th week, WRW was observed again in some cities in Hunan Province and after nine weeks, all 14 cities (prefectures) in Hunan Province had become green, indicating that the outbreak in Hunan Province has been completely controlled. In Shenzhen City and Xiamen City, although the epidemic occurred later than in the Hunan Province, the outbreak occurred within a short time. In contrast to the other three regions (Hunan province, Shenzhen City and Xiamen City), the HFMD outbreak appeared late in the Chuxiong Prefecture, in the spring or autumn. The outbreak of HFMD in two time periods required shorter time in spring to summer than in autumn to winter. For example, it only took 3 weeks (from the 15th week to the 17th week) in Hunan Province in spring and summer, and all areas in the province had turned red, but it took 8 weeks (from the 34th week to the 41st week) in autumn and winter. HFMD spread faster in the coastal cities such as Shenzhen City, and Xiamen City. However, from autumn to winter, there was no signal of HFMD outbreak in Jilin Province and Longde County.

## Discussion

In this study, LDE was used to study the early warning thresholds of HFMD cases over the years in Hunan Province, Jilin Province, Shenzhen City, Xiamen City, Chuxiong Prefecture, Yunxiao County, and Longde County. All models were tested for goodness of fit, and the results showed that more than 90% of the *R*^2^ obtained were statistically significant, and the model had good applicability.

Xing et al. analyzed the HFMD epidemic in the country and found that the prevalence time of HFMD in southern China and northern China is obvious[30]. HFMD in China has an annual incidence, with an obvious periodicity and time aggregation. In our six study regions, the burden of HFMD was high, and the early warning of HFMD in spring and autumn in the seven regions where we conducted an early warning threshold study was heterogeneous. In Fig 3, the northern region had a high incidence in summer, which peaked in June every year, while the southern region had two annual peaks. The first peak value occurred in May while the second occurred in September-October, which was consistent with the findings of Wang et al[30,34,35]. Therefore, our findings can be used as an epidemiological reference for similar studies in other areas. There are seasonal differences in the incidence characteristics of HFMD between different regions; therefore, when formulating prevention and control measures, it is necessary to control HFMD outbreak according to the actual situation. For example, in the southern region, prevention and control measures should be proposed in preparation for the spring and autumn seasons every year, while in the northern region, the prevention and control should be mainly in the summer.

Due to China’s vast territory and diverse environment, latitude is not the only factor affecting the spread of HFMD [34,36–38]. Based on a spatial autocorrelation analysis, Wang et al. found that the hotspots of HFMD in China were mainly distributed in the districts, counties and urban-rural junctions around the provincial capitals. These may have been related to population densities, economic conditions, and cross-infections among the population. Our findings revealed that HFMD outbreaks were more common and had a higher incidence in coastal cities. This phenomenon indicates that the presence of pathogenic virus causing HFMD may be related mainly to meteorological conditions such as the temperature, rainfall, and relative humidity of the affected area. Studies have also shown that the specific times of the incidence peak in the northern and southern regions are slightly different, and mainly affected by geographical location; the lower the general latitude, the earlier the appearance of the incidence peak [28,35,39–41].

Overall, this disease seems to spread more easily in spring than in autumn, but our research, indicated that the disease outbreaks in autumn in several coastal cities occurred much faster, such as the HFMD outbreaks that occurred within three weeks in all the counties affiliated to Shenzhen City and Xiamen City (Fig 13B). Furthermore, the diffusion rules in spring and autumn warnings are different, suggesting an explanation for the difference in transmission modes between the two seasons. In the future, an in-depth research is necessary in this area. At present, although the epidemiological characteristics of HFMD have been reported, the need for more descriptive studies remain.

In future studies, our research will be focused on the transmissibility of HFMD. Exploration of HFMD transmission dynamics and the effects of intervention measures are needed to better analyze the disease burden and propose prevention and control measures. Furthermore, a model that takes seasonal factors into account to calculate the transmission dynamics of HFMD in addition to a model that incorporates the effects of interventions, should be developed. The prevention and control measures of oral diseases can provide a more accurate basis to better prevent the spread of HFMD.

### Limitations

The incidence of reported HFMD and the number of cases were affected by the large difference in the reporting rate of HFMD in different regions of China. The LDE requires the data to be distributed symmetrically, but the actual data were not. Although our *R*^2^ fitted effect was good, the data from the actual data was difficult to fully idealize in the left and right symmetric distributions, resulting in some abnormal warning signals being reported, for example, the RWW was too late in the second peak in 2013 and the first in 2014. We aim to introduce the generalized LDE for the data analysis in future studies.

## Conclusions

The results obtained from Hunan Province, Jilin Province, Shenzhen City, Xiamen City, Chuxiong Prefecture, Yunxiao County, and Longde County, have indicated that the spread of HFMD in China remains extensive. The burden of HFMD in the seven regions is high, and the incidence of HFMD in the southern region is higher than that in the northern region. The early warning of HFMD in the seven regions is heterogeneous, with the northern regions having a high incidence in summer to autumn and the southern regions in spring to summer and autumn to winter. Moreover, the duration of early warning is shorter in spring to summer (6–10 weeks) than in autumn to winter (6–15 weeks), signifying a faster disease spread in spring to summer than in autumn to winter in the southern regions. Our research analyzed the epidemic cycle of HFMD; used LDE to analyze the characteristic of change in the epidemic rate in each epidemic cycle, from the slow to the fast phases; and calculated the EAW for each set of data to determine the RWW. These results support the need for the development of HFMD prevention strategies by the Center for Disease Control and Prevention staff at different time points in different regions in China.

## Supporting information

S1 Table The range of weeks for showing the median of the seven regions. (DOCX)
