# Supplementary material for: Early warning of hand, foot, and mouth disease transmission: A modeling study in mainland, China
Source: PLoS Negl Trop Dis. 2021 Mar 24;15(3):e0009233. doi: 10.1371/journal.pntd.0009233 (PMC8021164; doi:10.1371/journal.pntd.0009233)
Supplement: S1 Table — (DOCX) [file pntd.0009233.s001.docx]

**Supplementary Materials**

**S1 Table. The range of weeks for showing the median of the seven regions.**

| Regions | Spring to Summer | | | | | | Autumn to Winter | | | | | |
| --- | --- | --- | --- | --- | --- | --- | --- | --- | --- | --- | --- | --- |
|  | EAW (week) | | | WEW (week) | | | EAW (week) | | | WEW (week) | | |
|  | Median | Min | Max | Median | Min | Max | Median | Min | Max | Median | Min | Max |
| Hunan Province | 16 | 15 | 18 | 24 | 22 | 25 | 37.5 | 36 | 41 | 45.5 | 39 | 48 |
| Changsha City | 16.5 | 15 | 19 | 24 | 22 | 26 | 38 | 35 | 41 | 47.5 | 39 | 49 |
| Zhuzhou City | 17 | 15 | 19 | 24.5 | 22 | 27 | 37.5 | 34 | 40 | 42.5 | 38 | 48 |
| Xiangtan City | 16.5 | 14 | 19 | 23 | 19 | 28 | 37.5 | 21 | 42 | 44 | 27 | 50 |
| Hengyang City | 16.5 | 15 | 20 | 23 | 20 | 26 | 34 | 23 | 39 | 39.5 | 27 | 48 |
| Shaoyang City | 17 | 14 | 18 | 23.5 | 21 | 25 | 39 | 36 | 41 | 47 | 41 | 48 |
| Yueyang City | 16 | 15 | 20 | 23.5 | 22 | 27 | 38 | 36 | 40 | 45 | 43 | 48 |
| Changde City | 16.5 | 15 | 20 | 23 | 20 | 26 | 39.5 | 38 | 43 | 46 | 45 | 51 |
| Zhangjiajie City | 16.5 | 13 | 19 | 22.5 | 17 | 26 | 41 | 36 | 42 | 49 | 41 | 50 |
| Yiyang City | 17 | 14 | 20 | 23.5 | 23 | 26 | 38 | 36 | 41 | 46 | 40 | 50 |
| Chenzhou City | 17 | 15 | 20 | 23.5 | 21 | 28 | 39 | 37 | 40 | 45.5 | 40 | 48 |
| Yongzhou City | 15 | 13 | 19 | 23.5 | 22 | 26 | 37 | 34 | 39 | 44 | 41 | 47 |
| Huaihua City | 16.5 | 16 | 18 | 23.5 | 22 | 26 | 40 | 36 | 42 | 46 | 41 | 49 |
| Loudi City | 15.5 | 14 | 18 | 23 | 20 | 26 | 39 | 36 | 41 | 46 | 40 | 48 |
| Xiangxi State | 16 | 13 | 17 | 21.5 | 19 | 25 | 41 | 36 | 43 | 49 | 43 | 50 |
| Jilin Province | 25 | 20 | 35 | 31 | 26 | 42 | - | - | - | - | - | - |
| Baicheng City | 24 | 20 | 33 | 31 | 25 | 48 | - | - | - | - | - | - |
| Baishan City | 24 | 6 | 35 | 34 | 19 | 52 | - | - | - | - | - | - |
| Baoan District | 27 | 19 | 31 | 37 | 25 | 40 | - | - | - | - | - | - |
| Jilin City | 27 | 19 | 31 | 37 | 25 | 40 | - | - | - | - | - | - |
| Liaoyuan City | 22 | 20 | 27 | 35 | 29 | 47 | - | - | - | - | - | - |
| Siping City | 25 | 21 | 30 | 35 | 28 | 41 | - | - | - | - | - | - |
| Songyuan City | 26 | 11 | 36 | 32 | 15 | 48 | - | - | - | - | - | - |
| Tonghua City | 23 | 11 | 31 | 33 | 17 | 43 | - | - | - | - | - | - |
| Yanbian City | 25 | 22 | 29 | 35 | 28 | 38 | - | - | - | - | - | - |
| Changchun City | 26 | 21 | 30 | 33 | 27 | 40 | - | - | - | - | - | - |
| Shenzhen City | 17.5 | 14 | 24 | 25.5 | 21 | 33 | 37 | 34 | 37 | 42 | 40 | 44 |
| Baoan District | 18 | 14 | 26 | 25 | 20 | 35 | 35 | 34 | 38 | 41 | 40 | 43 |
| Futian District | 16.5 | 14 | 24 | 24.5 | 21 | 31 | 36 | 30 | 39 | 41 | 37 | 45 |
| Longgang District | 17 | 14 | 24 | 24.5 | 21 | 32 | 36 | 35 | 38 | 41 | 40 | 45 |
| Luohu District | 18 | 14 | 20 | 24 | 21 | 27 | 36 | 33 | 38 | 41.5 | 38 | 45 |
| Nanshan District | 18.5 | 14 | 24 | 25 | 20 | 34 | 37 | 34 | 40 | 41 | 40 | 44 |
| Yantian District | 17 | 14 | 29 | 25.5 | 21 | 37 | 36.5 | 35 | 42 | 42.5 | 40 | 46 |
| Xiamen City | 18 | 15 | 19 | 24 | 21 | 28 | 36 | 35 | 39 | 42 | 40 | 45 |
| Haicang District | 17 | 16 | 19 | 24 | 20 | 26 | 37 | 35 | 38 | 42 | 39 | 45 |
| Huli District | 16 | 15 | 20 | 23 | 21 | 28 | 37 | 36 | 38 | 41 | 41 | 45 |
| Jimei District | 17 | 15 | 19 | 23 | 20 | 26 | 36 | 34 | 39 | 41 | 38 | 45 |
| Siming District | 16 | 16 | 20 | 23 | 22 | 27 | 37 | 35 | 38 | 42 | 40 | 44 |
| Tongan District | 17 | 14 | 19 | 24 | 21 | 26 | 38 | 35 | 40 | 42 | 41 | 47 |
| Xiangan District | 17 | 14 | 21 | 24 | 21 | 27 | 37 | 35 | 40 | 42 | 39 | 44 |
| Chuxiong Prefecture | 18 | 15 | 21 | 28 | 23 | 31 | 43.5 | 38 | 45 | 47 | 43 | 52 |
| Yunxiao County | 17 | 12 | 29 | 23 | 18 | 37 | 40 | 33 | 47 | 47 | 39 | 51 |
| Longde County | 21 | 15 | 26 | 31 | 20 | 34 | - | - | - | - | - | - |
